# Treatment of *Mycoplasma genitalium*. Observations from a Swedish STD Clinic

**DOI:** 10.1371/journal.pone.0061481

**Published:** 2013-04-08

**Authors:** Carin Anagrius, Britta Loré, Jørgen Skov Jensen

**Affiliations:** 1 Department of Venereology, Falu lasarett, Falun, Sweden; 2 Department of Clinical Microbiology, Falu lasarett, Falun, Sweden; 3 Statens Serum Institut, Microbiology and Infection Control, Sexually Transmitted Infections, Research and Development, Copenhagen, Denmark; Ghent University, Belgium

## Abstract

**Objectives:**

To evaluate therapy for *Mycoplasma genitalium* infection with doxycycline or azithromycin 1 g compared to five days of azithromycin (total dose 1.5 g).

**Methods:**

A retrospective case study was performed among patients attending the STD-clinic in Falun, Sweden 1998–2005. All patients with a positive PCR test for *M. genitalium* were routinely offered a test of cure (toc). Response to doxycycline for 9 days, azithromycin 1 g single dose and extended azithromycin (500 mg on day 1 followed by 250 mg o.d. for 4 days) was determined. In patients with treatment failure after azithromycin, macrolide resistance was monitored before and after treatment. Furthermore, the rate of macrolide resistance was monitored for positive specimens available from 2006–2011.

**Results:**

The eradication rate after doxycycline was 43% (48% for women and 38% for men), for azithromycin 1 g 91% (96% for women and 88% for men) and for extended azithromycin 99% (100% for women and 93% for men). Macrolide resistance developed in 7/7 examined (100%) of those testing positive after azithromycin 1 g, but in none of those treated with extended azithromycin. Macrolide resistance before treatment increased from 0% in 2006 and 2007 to 18% in 2011.

**Conclusions:**

These findings confirm the results from other studies showing that doxycycline is inefficient in eradicating *M. genitalium*. Although azithromycin 1 g was not significantly less efficient than extended dosage, it was associated with selection of macrolide resistant *M. genitalium* strains and should not be used as first line therapy for *M. genitalium*. Monitoring of *M. genitalium* macrolide resistance should be encouraged.

## Introduction

*Mycoplasma genitalium* has become a well-established sexually transmitted infection (recently reviewed in [1]) and the main focus is now on therapy and complications. It was first isolated in 1980 from two of 13 men with non-gonococcal urethritis (NGU) [2], [3]. However, due to the extremely fastidious nature of the bacterium, clinical studies were not feasible until the advent of sensitive and specific polymerase chain reaction (PCR) methods [4]. Several publications have shown association between *M. genitalium* and urethritis in men and urethritis, cervicitis, endometritis, and pelvic inflammatory disease in women [1], [5]–[9]. In comparison with other sexually transmitted infections there is less information about the global prevalence of *M. genitalium*. The prevalence of *M. genitalium* in men with non-chlamydial NGU (NCNGU) ranges from 10% to 45% [1] and in the general population from 1% to 3,3% [10], [11].

*M. genitalium*, like all other mycoplasmas has no rigid cell wall [12] and therefore beta-lactam antibiotics and other antibiotics acting on the cell wall are not active. In vitro studies suggested that *M. genitalium* is highly susceptible to macrolides, particularly to azithromycin, but that it has reduced susceptibility to tetracyclines and older quinolones [13]. Ketolides [14] and some new fluoroquinolones such as moxifloxacin have sufficiently low MIC in vitro [13] and moxifloxacin is currently the most commonly used second-line antibiotic in patients failing azithromycin treatment. Side effects, cost, and the risk of selection of resistant strains are limiting factors for the use of moxifloxacin.

There are currently no evidence-based guidelines specifically for the treatment of *M. genitalium* infection. Clinical experience and observational studies [15]–[18] demonstrated insufficient microbiological and clinical cure with tetracyclines and levofloxacin whereas azithromycin seemed to be more efficacious. Only three randomized trials have compared doxycycline with azithromycin 1 g single dose. Two concluded that azithromycin was clearly superior eradicating 87% [19] and 67% [20] of the infections, respectively. In contrast, a recently published double-blind, randomized trial found no significant difference in cure rates between single dose azithromycin and doxycycline, both eradicating <40% of the infections [21]. In general, treatment of an STI should usually aim at a treatment efficacy of at least 95% in order to be clinically useful [22], and this is far from the efficacy seen with the 1 g single dose of azithromycin.

The US CDC STD Treatment Guideline [23] recommends azithromycin 1 g as first-line therapy for uncomplicated *M. genitalium* infection. In many countries, but not in Sweden, azithromycin 1 g is also first line therapy for chlamydia and NGU of unknown aetiology.

Persistence of *M. genitalium* is associated with recurrent or persistent NGU. Bradshaw *et al*. [17] showed that 91% of patients with persistent *M. genitalium* infection experienced persistent symptoms compared to 17% of patients in whom *M. genitalium* was eradicated. Looking at men with treatment failure after tetracycline, 41% of men returning with recurrent or persistent urethritis were *M. genitalium* positive [24].

The objective of the present case study was to compare the efficacy of doxycycline 200 mg given on day 1 followed by 100 mg day 2–9 versus orally administered azithromycin 1 g as a single dose, and extended azithromycin therapy, 500 mg orally on day 1 followed by 250 mg orally daily on day 2–5. The doxycycline dosage is the recommended treatment for uncomplicated chlamydial infections and NGU in Sweden. The extended azithromycin dosage which has become the standard treatment for *M. genitalium* in Sweden was based on the commonly used dosage for *M. pneumoniae* infections in Sweden. Furthermore, we aimed to follow the development of macrolide resistance in our local population during recent years.

## Methods

### Study design

This study was performed as a retrospective case-study of patients attending the Department of Venereology, Central Hospital, Falun, Sweden with a diagnosis of *M. genitalium* infection confirmed by PCR. Case files were reviewed in individuals who tested positive between January 1998 and July 2005. Long term follow up was conducted until December 2009.

In order to study the level of macrolide resistance, remnants of available *M. genitalium* positive specimens from July 2006 through December 2011 stored at −20°C were examined for mutations mediating macrolide resistance.

### Study population

Specimens for *M. genitalium* PCR were taken from patients with symptoms or signs of genital tract infection, especially symptoms and signs of urethritis and cervicitis in the patient or partner, in sexual contacts to *M. genitalium*-infected patients and also in patients suspected of complications i.e. irregular bleeding, lower abdominal pain and epididymitis. Between January 2003 and July 2004, all patients were routinely tested for *M. genitalium* regardless of symptoms

### Clinical procedures

A detailed medical history was obtained, and physical examination performed according to standard procedures as previously described [8]. Signs and symptoms were extracted from the records for the present study. Patients were regarded as having symptoms of urethritis if they complained of dysuria or urgency and for males if they had noticed a discharge. In females, discharge was regarded as a symptom of cervicitis. Patients returning for follow-up were asked about resolution of symptoms, compliance with treatment and condom use. Reinfection was considered likely based on the sexual history when unprotected sex was reported with a new partner or an untreated or inadequately treated partner. When samples were available, *M. genitalium* MgPa SNP typing was performed at previously described [25]

From January 1998 through December 2002 specimens for *M. genitalium* and chlamydia were collected with swabs from the urethra in men and from the urethra and the cervix in women and transported in 2 SP medium.

From January 2003 men provided first voided urine (10 ml), and from women a swab from the cervix was collected and transported in first voided urine (10 ml).

Microscopy of methylene blue stained specimens was routinely performed on urethral smears from both women and men and on cervical smears from women. Smears were examined at 1000× magnification for the presence of polymorphonuclear cells (PMNLs). Vaginal wet smears were examined for the presence of PMNLs, clue cells, *Trichomonas vaginalis* and yeasts. Urethritis was defined as ≥5 PMNLs in urethral smears and cervicitis as ≥30 PMNLs/high power field in cervical smears *or* more PMNLs than epithelial cells in the wet smear.

All participants were asked to abstain from sexual intercourse during treatment and from unprotected sex until test of cure (toc). Follow-up for toc was scheduled at earliest four weeks after start of treatment. Time for toc was registered as 4–12 weeks and 13–52 weeks after start of treatment. Patients with toc after more than 52 weeks were excluded. Microbiological failure was defined as a positive PCR-test for *M. genitalium* at toc. Clinical failure was defined as the presence of symptoms or signs of urethritis or cervicitis.

From 1998–2003 primary treatment for urethritis and cervicitis, before the aetiology was known, was doxycycline 200 mg day 1 and 100 mg days 2–9. From July 2003 to July 2005 azithromycin 1 g was given as a single dose as primary treatment.

Azithromycin 500 mg on day 1 followed by 250 mg o.d. on days 2–5 (extended azithromycin) was given to *M. genitalium* positive patients when no other treatment was given initially and to patients when a partner had a documented *M. genitalium* infection. Secondary extended azithromycin was given when microbiological failure after doxycycline treatment was documented.

In those testing positive at toc following azithromycin treatment, and when reinfection was considered unlikely, moxifloxacin 400 mg o.d. for 7 days was prescribed.

**Figure 1:**
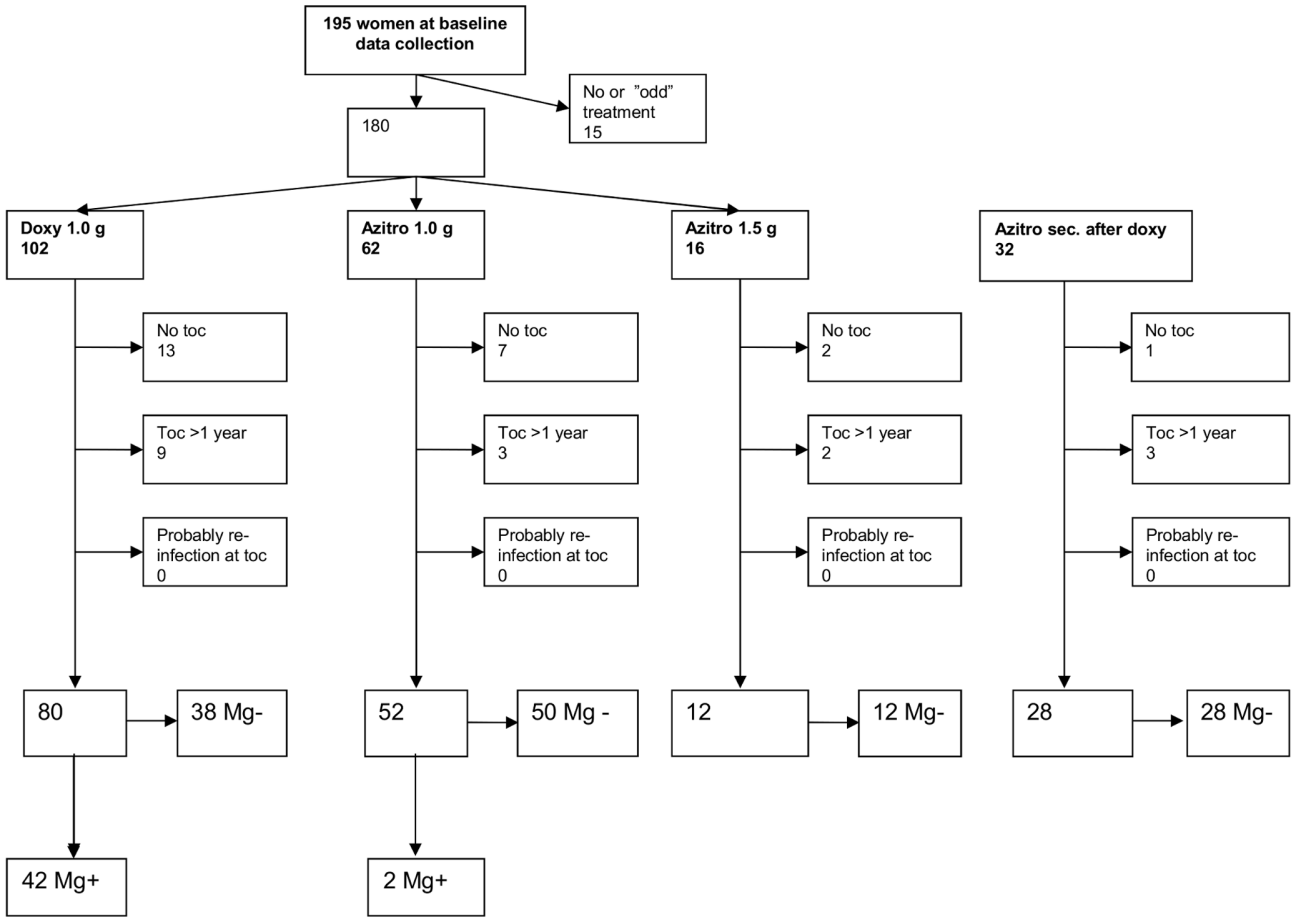
Flow-chart showing reasons for exclusion and treatment outcomes for women according to antibiotic treatment.

**Figure 2:**
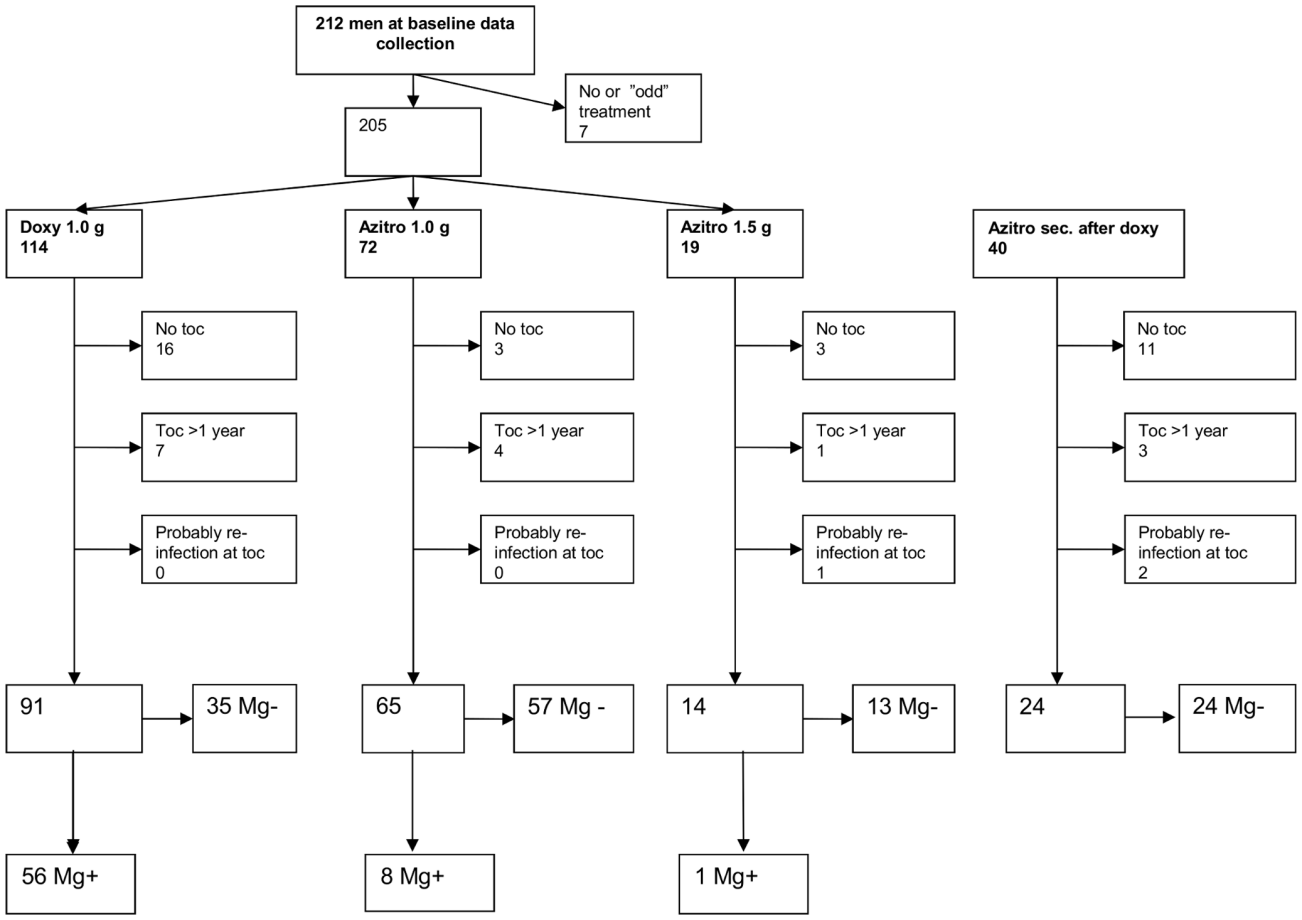
Flow-chart showing reasons for exclusion and treatment outcomes for men according to antibiotic treatment.

Patients were excluded both from the study of microbiological and clinical cure if they had no toc, late toc (>52 weeks), no or other treatment than doxycycline, azithromycin and moxifloxacin, or if reinfection was considered likely. Additionally, patients co-infected with other STIs initially or at toc were excluded from evaluation of clinical cure. Inclusion, assignment to treatment, and reasons for exclusion is shown in figures 1 and 2.

From 11 patients testing positive for *M. genitalium* after treatment with azithromycin, initial and toc samples were available from nine. These were analysed for macrolide resistance mediating mutations (see below).

Long term treatment efficacy was evaluated for patients having completed a toc with a negative *M. genitalium* result and returning to the clinic one year or more after toc.

### Laboratory procedures

PCR for *C. trachomatis* was performed using the Roche Amplicor kit according to the manufacturer's instructions. *M. genitalium* was detected by PCR with gel-based detection using the MgPa-1/MgPa-3 primer-set [4]. All positive results were confirmed using primers amplifying the *M. genitalium* 16S rRNA gene [8], [26]. *N. gonorrhoeae* was detected by culture.

For detection of macrolide resistance mediating mutations in region V of the *M. genitalium* 23S rRNA gene, extracted DNA or remnants of the original specimen was shipped to Copenhagen and analysed by DNA sequencing as previously described [27]. Analysis for missense mutations in the L4 and L22 ribosomal protein genes was not conducted as all *M. genitalium* strains isolated from patients failing treatment with macrolides have carried mutations in region V of the 23S rRNA gene explaining the resistance. Specimens with macrolide resistance mediating mutations from 2008 through May 2011 as well as a random selection of 15 specimens without mutations and strains occurring in 2011 with the relatively uncommon mutation A2058C (*E. coli* numbering) were subjected to the MgPa SNP-typing system previously described [25] in order to demonstrate a clonal spread of macrolide resistant *M. genitalium* strains.

### Ethical considerations

Ethical approval and exemption for obtaining informed consent was obtained from the Uppsala Research Ethical Committee (journal no. 2010/429) in accordance with Swedish law on quality development projects. The risk that the integrity of any individual should be compromised was estimated as minimal in view of the fact that the study was performed on data anonymised after extraction from the patient records. No actions, treatments or visits were changed from routine patient management.

### Statistical analysis

Fischer`s exact test was used for statistical test of proportions.

## Results

During the study period between January 1998 and July 2005, a total of 8450 tests for *M. genitalium* were analysed, 4095 from women and 4355 from men. Of those, 407 (4.8%) patients, 195 (4.8%) women and 212 (4.9%) men were positive. For comparison, 11419 tests for chlamydia were analysed in the same period, 5147 women and 6255 men. Of those, 675 (5.9%) patients were positive, 280 (5.4%) women and 395 (6.3%) men.

The prevalence of *M. genitalium* during the screening period from January 2003 through June 2004 was 3.7% and 4.2% for women and men, respectively, and in the remaining period with more targeted testing, the prevalence was 5.2% both for women and men.

### Evaluation of microbiological cure

In the study of microbiological cure, 314 (82%) of 385 patients with primary treatment were followed-up within 52 weeks, 144 women and 170 men. A total of 93 patients, 51 women and 42 men, were excluded as explained in figures 1 and 2. Of the 314 patients followed up within 52 weeks, 255 (81%) were followed up within 12 weeks after initiation of treatment.

Patients failing treatment with doxycycline were treated with extended azithromycin 1.5 g as second-line treatment, and this regimen was given to 72 patients.

### Doxycycline

Doxycycline for nine days was given to 216 patients, 102 women and 114 men. One hundred and seventy one (79%), 80 (78%) women and 91 (80%) men, attended for follow up. At the time of toc, 73 (43%), 38 (46%) women and 35 (38%) men were *M. genitalium* PCR negative (Table 1).

**Table 1:** Microbiological cure of patients at test of cure (toc) according to treatment.

	Doxycycline		Azithromycin 1 g single dose		Azithromycin 1.5 g extended dose		Azithromycin 1.5 g extended dose, secondary treatment	
	n	*M. genitalium* eradicated at toc	n	*M. genitalium* eradicated at toc	n	*M. genitalium* eradicated at toc	n	*M. genitalium* eradicated at toc
All patients	171	73 (43%)	117	107 (91%)***	26	25 (96%)***	52	52 (100%)***
Male patients	91	35 (38%)	65	57 (88%)***	14	13 (93%)***	28	28 (100%)***
Female patients	80	38 (46%)	52	50 (96%)***	12	12 (100%)***	24	24 (100%)***

Patients given azithromycin extended dose as secondary treatment had received doxycycline as primary treatment.

*** p<0.0005 compared to *M. genitalium* eradication after doxycycline

### Azithromycin

Azithromycin 1 g as a single dose was given as primary treatment to 134 patients (62 women and 72 men). One hundred and seventeen (88%), 52 (84%) women and 65 (90%) men attended for follow up. At time of toc 107 (91%), 50 (96%) women and 57 (88%) men were *M. genitalium* PCR negative (Table 1).

Extended azithromycin 1.5 g was given to 35 patients as primary treatment (16 women and 19 men). Twentysix (74%), 12 (75%) women and 14 (74%) men attended for follow up. At time of toc 25 (96%), 12 (100%) women and 13 (93%) men were *M. genitalium* PCR negative (Table 1). The single man failing extended azithromycin was infected with a strain of *M. genitalium* that carried a macrolide resistance mediating sequence present before initiation of treatment.

Extended azithromycin 1.5 g as second-line treatment, i.e. after having a positive toc after treatment with doxycycline, was given to 72 patients, 32 women and 40 men. A total of 52 (72%), 28 (88%) women and 24 (60%) men attended for follow up. All 52 had a negative *M. genitalium* PCR test at toc (Table 1).

### Moxifloxacin

Moxifloxacin 400 mg o.d. for 7 days was given to 10 patients with a positive toc after treatment with azithromycin. Nine (90%) of the patients had a toc. All 9 had negative *M. genitalium* PCR results after treatment.

### Comparison of microbiological cure

Azithromycin 1 g single dose and 1.5 g extended dosage had a significantly higher microbiological cure-rates then doxycycline (Table 1) when all patients were compared (p<0.0001 for both). For men and women separately, all comparisons between doxycycline and azithromycin were also significantly in favour of azithromycin (p<0.0005). No statistically significant difference in the cure rates between the two azithromycin dosages when given as primary treatment could be found, to some extent due to the small number of patients given 1.5 g as the primary treatment (Table 1).

### Symptoms and signs

#### Women

For female patients with information available both at inclusion and at toc, symptoms before treatment with doxycycline, azithromycin 1 g and 1.5 g was reported in 85, 57, and 75% of the women, respectively. For women with symptomatic infection at inclusion, symptoms decreased for those microbiologically cured to 38, 40, and 22%, respectively (Table 2). Significantly fewer of those experiencing microbiological cure after doxycycline had symptoms (10 of 26, 38%) than those with treatment failure (21 of 29, 72%) (p = 0.01) (Table 2). Too few women failed treatment with azithromycin to make meaningful comparisons. No significant difference in the proportion of patients becoming asymptomatic after eradication of *M. genitalium* by the three different treatments could be found.

**Table 2:** Persistence of symptoms in patients at test of cure (toc) among patients having symptoms before treatment according to treatment and persistence or eradication of *M. genitalium*.

	Doxycycline				Azithromycin 1 g single dose				Azithromycin 1,5 g extended dose			
	*M. genitalium* persistence		*M. genitalium* eradicated		*M. genitalium* persistence		*M. genitalium* eradicated		*M. genitalium* persistence		*M. genitalium* eradicated	
	n	Symptoms at toc	n	Symptoms at toc	n	Symptoms at toc	n	Symptoms at toc	n	Symptoms at toc	n	Symptoms at toc
All patients	70	50 (71%)	48	20 (42%)^**^	8	0 (0%)	58	15 (26%)	1	1 (100%)	18	2 (11%)†
Male patients	41	29 (71%)	22	10 (45%)	7	4 (57%)	33	5 (15%)* †	1	1 (100%)	9	0 (0%)†
Female patients	29	21 (72%)	26	10 (38%)*	1	0 (0%)	25	10 (40%)	0	0	9	2 (22%)

* p<0.05 ^**^p<0.005 for comparisons between patients with *M. genitalium* persistence or eradication within the same treatment group.

† p<0.05 for comparisons between symptoms among patients with eradication after doxycycline treatment compared with azithromycin

Microscopic signs of urethritis and/or cervicitis were found in 98, 91 and 100% at inclusion and among women with signs at inclusion, signs decreased to 53, 50, and 30% for those microbiologically cured (Table 3). Significantly fewer of those experiencing microbiological cure after doxycycline had signs at toc (17 of 32, 53%) than those with treatment failure (27 of 31, 87%) (p = 0.004) (Table 3). Too few women failed treatment with azithromycin to make meaningful comparisons. No significant difference in the proportion of patients becoming microscopically negative after eradication of *M. genitalium* by the three different treatments could be found.

**Table 3:** Persistence of microscopic signs of urethritis and cervicitis in patients at test of cure (toc) among patients having signs before treatment according to treatment and persistence or eradication of *M. genitalium*.

	Doxycycline				Azithromycin 1 g single dose				Azithromycin 1,5 g extended dose			
	*M. genitalium* persistence		*M. genitalium* eradicated		*M. genitalium* persistence		*M. genitalium* eradicated		*M. genitalium* persistence		*M. genitalium* eradicated	
	n	Signs at toc	n	Signs at toc	n	Signs at toc	n	Signs at toc	n	Signs at toc	n	Signs at toc
All patients	82	76 (93%)	59	34 (57%)^***^	9	6 (67%)	71	31 (44%)	1	1 (100%)	20	5 (25%)†
Male patients	51	49 (96%)	27	17 (63%)^***^	7	5 (71%)	33	12 (36%)†	1	1 (100%)	10	2 (20%)†
Female patients	31	27 (87%)	32	17 (53%)**	2	1 (50%)	38	19 (50%)	0	0	10	3 (30%)

** p<0.005 ^***^p<0.0005 for comparisons between patients with *M. genitalium* persistence or eradication within the same treatment group.

† p<0.05 for comparisons between signs among patients with eradication after doxycycline treatment compared with azithromycin.

#### Men

For male patients with information available both at inclusion and at toc, symptoms before treatment with doxycycline, azithromycin 1 g and 1.5 g were reported in 78, 70, and 86%, respectively. For men with symptomatic infection at inclusion, symptoms decreased to 45, 15, and 0% for those microbiologically cured (Table 2). Fewer of those experiencing microbiological cure after doxycycline had symptoms (10 of 22, 45%) than those with treatment failure (29 of 41, 71%), however, this difference was not statistically significant (p = 0.058) (Table 2). Too few men failed treatment with azithromycin to make meaningful comparisons. A significantly larger proportion of men successfully treated with azithromycin became asymptomatic at toc than of those treated with doxycycline; this was the case for both those treated with the 1 g single dose (p = 0.018) and those treated with the extended 1.5 g dosage scheme (p = 0.014) (Table 2). No difference in the proportion of patients becoming asymptomatic after eradication of *M. genitalium* by the two azithromycin dosages could be found.

Urethritis defined by microscopy was present in 99, 91, and 92% before treatment, and among men with signs at inclusion, signs decreased to 63, 36, and 20% of those microbiologically cured at toc (Table 3). Significantly fewer of those experiencing microbiological cure after doxycycline had signs at toc (17 of 27, 63%) than those with treatment failure (49 of 51, 94%) (p = 0.0003). Too few men failed treatment with azithromycin to make meaningful comparisons. A significantly larger proportion of men successfully treated with azithromycin had no signs at toc than of those treated with doxycycline; this was the case for both those treated with the 1 g single dose (p = 0.046) and those treated with the extended 1.5 g dosage scheme (p = 0.027) (Table 3). No difference in the proportion of patients without signs after eradication of *M. genitalium* by the two azithromycin dosages could be found.

### Macrolide resistance

Results from the 10 patients testing positive after treatment with azithromyzin 1 g were evaluated for macrolide resistance mediating mutations. Two pre-treatment samples were missing but the post treatment samples carried resistance mutations. Samples from one patient were inconclusive due to lack of sufficient amounts of *M. genitalium* DNA. Samples from the remaining 7 patients which could be completely evaluated showed a susceptible genotype before treatment, whereas all carried resistance mediating mutations after treatment. Thus, macrolide resistance was induced in 7/7 (100%) evaluable patients failing azithromycin 1 g single dose.

The single male patient failing treatment with extended 1.5 g azithromycin carried a resistance mediating mutation in the pre-treatment specimen explaining the treatment failure. Thus, if the three inconclusive pre- and post-treatment specimen sets are excluded, 7 of 114 patients (6%) treated with azithromycin 1 g single dose developed resistance during treatment compared to none of 25 patients treated with the extended 1.5 g dose as primary treatment (excluding the patient with pre-existing resistance) (p = 0.24) and none of the 52 patients treated with the extended 1.5 g dose as secondary treatment.

**Figure 3:**
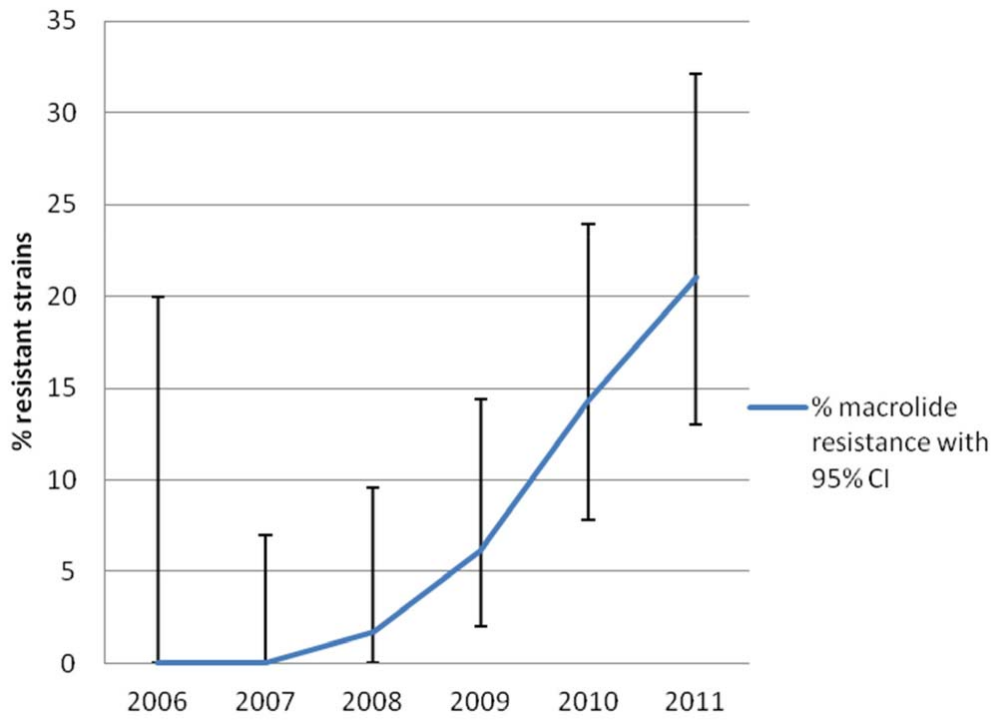
Increase in the proportion of patients infected with *M. genitalium* strains carrying macrolide resistance mediating mutations in region V of the 23S rRNA gene. Error-bars mark the 95% confidence interval (95% CI) for the estimate.

From July 2006 until the end of 2011, a total of 566 *M. genitalium* positive samples from 509 patients were received in the diagnostic laboratory. Among these, 431 samples (73%) were available for evaluation of macrolide resistance mediating mutations, and 408 (95%) contained sufficient amounts of *M. genitalium* DNA to be analysed. From 2006 through 2007 no macrolide resistance was detected, in 2008 a single sample carried the macrolide resistance determinant (<2%) whereas the proportion of resistant samples increased to 6% in 2009, 14% in 2010 and 21% in 2011(Fig. 3).

**Figure 4:**
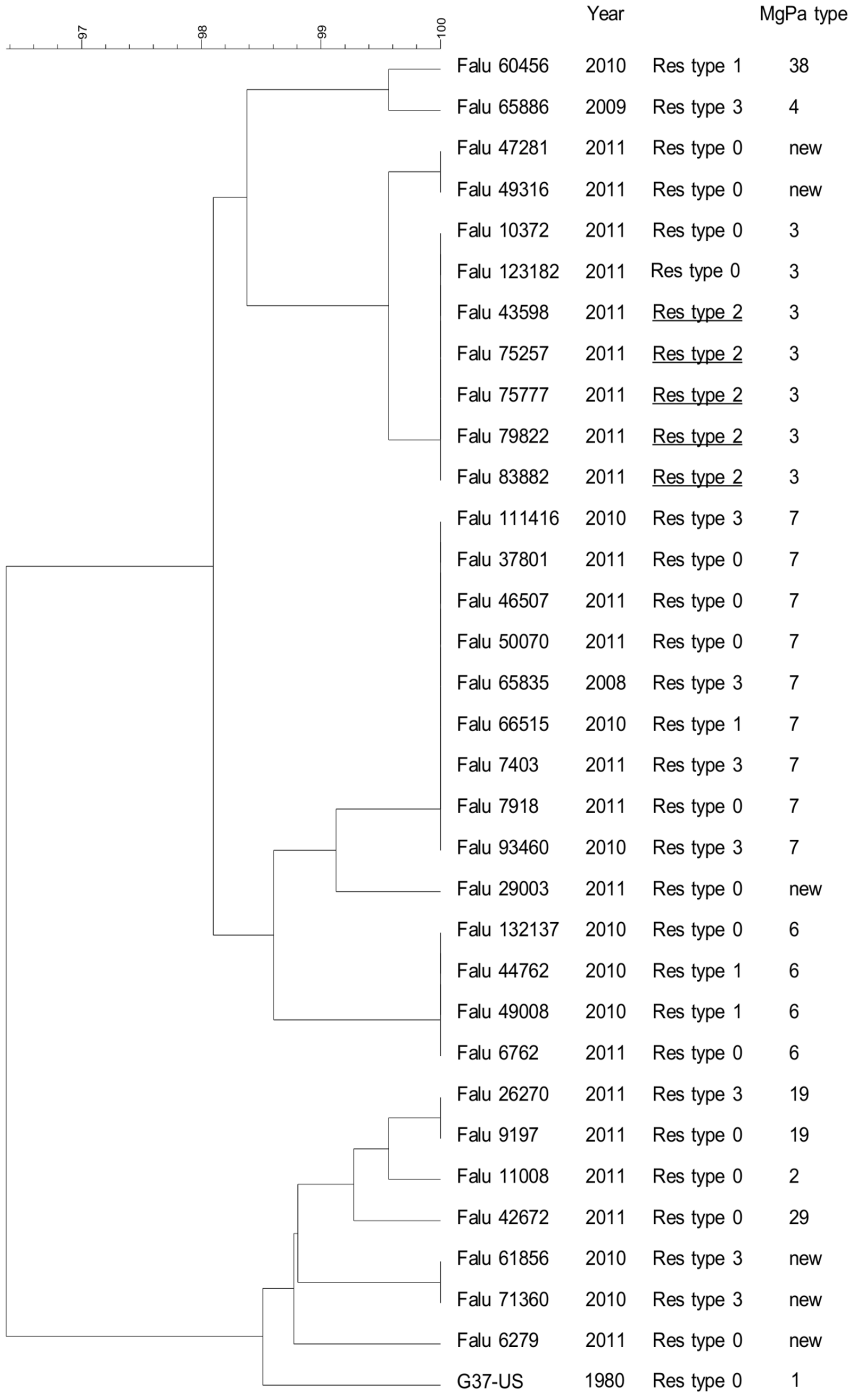
Dendrogram showing clustering of MgPa (MG192) gene sequences from patients with and without macrolide resistance mediating mutations in region V of the 23S rRNA gene. The sequence of the *M. genitalium* G37 type-strain is included for reference. The year of specimen collection, the resistance type, and the MgPa type is given for each specimen. Resistance type 0 is the wild-type macrolide susceptible genotype; resistance type 1 is A2059G, resistance type 2 is A2058C, and resistance type 3 is A2058G.

DNA typing of specimens carrying the resistance mediating mutations did not suggest that the increase was due to clonal spread of macrolide resistant *M. genitalium* strains in 2009 and 2010, but in 2011, 5 patients were identified with the relatively uncommon mutation A2058C (*E. coli* numbering) and all of these patients were infected with the same clone, suggesting a spread in the community due to treatment failure (Fig. 4). If this clonal spread was excluded from the calculation, the resulting proportion of resistant samples in 2011 would be 16%, more comparable to the figure from 2010.

### Long term follow up

A total of 168/257 (65%) patients having a negative toc, 89/128 (69%) women and 79/129 (61%) men (figures 1 and 2) had at least one new test for *M. genitalium* at least one year after first negative toc. The patients were initially microbiologically cured after doxycycline in 73/257 (28%), azithromycin 1 g 108/257 (42%) and azithromycin extended dosage in 77/257 (30%). At late follow up, 164 of the168 patients with long-term follow-up had a negative test for *M. genitalium*. Sexual history and strain typing in the 4 patients with a positive test suggested a newly acquired infection. One was positive for macrolide resistance in the first but not in the second sample and DNA typing was performed on samples from first and second infection in 3 of the 4, and all showed different DNA types further indicating that the second infection was newly acquired and not due to persistence of an incompletely eradicated *M. genitalium* infection.

## Discussion

These clinical observations provide further evidence that doxycycline is less efficient than azithromycin in eradicating *M. genitalium* from the urogenital tract. Both the 1 g single dose and the extended treatment with 1.5 g azithromycin were significantly more efficient than doxycycline. The cure-rate for azithromycin 1 g single dose was not significantly different from that of the extended treatment. However, we found that macrolide resistance was induced in 7 of 7 (100%) patients with persistent infection after azithromycin 1 g in cases where this could be evaluated. The single patient failing extended azithromycin was infected with a macrolide resistant strain of *M. genitalium* already before initiation of treatment and consequently, the treatment failure was not surprising. Thus, we showed that the risk of developing resistance during treatment was higher for the 1 g single dose regimen than for the 1.5 g extended dose regimen, although this result did not reach statistical significance due to the small sample size. Furthermore, as three out of 10 specimen-sets could not be evaluated, the risk of development of resistance during azithromycin 1 g single dose treatment may indeed be underestimated. The notion that the extended azithromycin treatment is less likely to induce or select for macrolide resistance is further supported by the finding that among the 52 patients treated with the 1.5 g dose after doxycycline treatment failure, no treatment failures due to macrolide resistance were detected. If the two dataset were combined, the difference in the rate for resistance development would actually reach statistical difference (p = 0.025 for pooled 1.5 g data). However, it may not be reasonable to pool the data, as it cannot be excluded that a previously failed treatment with doxycycline could decrease the likelihood for selection of macrolide resistance during a subsequent azithromycin treatment, although the opposite may seem more likely if it was due to genuine doxycycline resistance. However, treatment failure after doxycycline therapy does not correlate with increased doxycycline MIC *in vitro* (Jensen, JS; unpublished), and therefore, our findings support the need for a randomised treatment trial comparing the 1 g single dose and the 1.5 g extended dose treatment. The findings strongly suggest that routine use of azithromycin 1 g single dose for NGU/cervicitis may lead to increased levels of *M. genitalium* resistance and are in good agreement with findings from Australia showing that in this setting, more than half of the treatment failures after azithromycin 1 g single dose were due to development of resistance during treatment [28].

In this study, we found an unexpected high cure rate of 91% after azithromycin 1 g. Different cure rates ranging from 40%–87% have been reported [18]–[21], [29] in studies from different countries. This may be a consequence of different rates of macrolide resistance in the populations. Interestingly, older studies tend to show a higher cure rate for azithromycin as exemplified by the three US randomised trials where the study by Mena et al. [19] included patients in 2002 to 2004 and found a cure rate of 87% after 1 g azithromycin single dose as compared to the trial by Schwebke *et al*. [20] including patients from November 2006 to April 2009 and reporting a cure rate of only 67%. Most recently, the study reported by Manhart *et al*. [21] included patients from 2007 to July 2011 and found a cure rate of only 40%, not different from that of doxycycline at 30%.

We found no primary macrolide resistance in specimens obtained before treatment in our population from 2006–2007, i.e. in the first two years after the patients for this case-study were included. However, a dramatic increase in the macrolide resistance level was observed in our area, particularly in 2010 and 2011 reaching 21% in 2011. This increase may be suggestive of a rapid development of macrolide resistance in Sweden. Recently, resistance rates as high as 40% in Denmark (Jensen, JS, unpublished) and even 100% in Greenland [30] have been observed. These differences in the prevalence of resistance are believed to be a reflection of different guidelines or traditions of treatment. The reason for the increase in macrolide resistance in our population is unclear, however. The clinical guidelines for treatment of *C. trachomatis* infections and NGU/cervicitis of unknown aetiology have not been changed, and doxycycline is still the preferred treatment in Sweden. An overall increase in the macrolide consumption for non-STIs could explain the increasing resistance, but this does not seem to be the explanation, since consumption figures for macrolides for outpatients have shown decreasing trends both for Sweden in general, and for Dalarna (the area under study) in particular (http://www.smi.se/amnesomraden/antibiotikaresistens/statistik-antibiotikaforsaljning/sverige-totalt/oppenvard/). For the age-group from 15–44 years, the total Swedish macrolide consumption decreased from 0.77 defined daily doses (DDD)/1000 inhabitants per day in 2001 to 0.29 DDD in 2010. Similar figures for Dalarna were 0.65 DDD in 2001 decreasing to 0.25 DDD in 2010. Also, macrolide resistance in *Streptococcus pneumoniae* has been largely constant between 2004 and 2012 with minor fluctuations between 4.4 and 7% (http://resnet.smi.se/ResNet/findAntibiotikum.jsp).

An introduction and subsequent spread of a macrolide resistant clone of *M. genitalium* could also explain the increase in resistance. However, as the macrolide resistance mediating mutations in 2010 were of three different types, this explanation is unlikely. Furthermore, when specimens with resistance mediating mutations from 2008 until the first part of 2011 were DNA typed using the MgPa SNP typing system [25] no apparent clustering was obvious. A few specimens with resistance mediating mutations did actually cluster together, but except for one pair of specimens, they always did so together with susceptible strains, suggesting that the DNA types were commonly present in the population. Consequently, DNA typing did not support the idea of a clonal spread. Thus, if influx of resistant *M. genitalium* strains was the explanation, this influx must have been from different sources. The pattern, however, changed in the later part of 2011 where an apparent spread of the uncommon A2058C mutation occurred among five patients, all having the same MgPa SNP type suggesting that on-going monitoring of macrolide resistance may be worthwhile.

Azithromycin in the extended dosage with 1.5 g had a cure rate of 96% in men and 100% in women in our material, figures similar to those in two other published treatment studies including patients mainly in Sweden [16], [18]. The single patient failing treatment with this dosage was infected with an *M. genitalium* strain with pre-existing resistance. However, a study from Norway [29] reported a cure rate of only 78% for this treatment regimen. This difference may reflect a widespread use of azithromycin 1 g single dose as primary treatment for *C. trachomatis* infections and syndromic NGU/cervicitis treatment in Norway, and, indeed, it has recently been suggested that the recommendations should be changed to avoid azithromycin 1 g single dose and to use doxycycline as the primary treatment [31]. In the present study, moxifloxacin was given as second line therapy only to 10 patients, and all of the nine patients returning for a toc were *M. genitalium* negative after treatment. However, oral moxifloxacin should not be used uncritically as it should be reserved for severe infections and where other antibiotics cannot be used or have failed. This recommendation was issued by the European Medicines Agency due to the very rare but serious adverse hepatic reactions that had been observed (http://www.emea.europa.eu/ema/index.jsp?curl=pages/medicines/human/referrals/Moxifloxacin/human_referral_000114.jsp&mid=WC0b01ac05805c516f). Furthermore, *M. genitalium* strains with high-level moxifloxacin and macrolide resistance have been isolated from patients failing treatment (Jensen, JS; unpublished) and mutations suspected of mediating high-level quinolone resistance have been shown to appear during treatment with the related quinolone gatifloxacin [32] suggesting that selection of resistance may readily result with more widespread use.

Our long term follow up of patients with apparent sufficient treatment could not confirm persistence or latency of *M. genitalium* infection after initial cure. We did this follow-up to address concerns that *M. genitalium* may persist for a prolonged period of time, despite appropriate treatment such as it has been shown in respiratory tract infections with the closely related *M. pneumoniae* [33].

Our data emphasize the need for readily available diagnostic tests for *M. genitalium* including tools to detect macrolide resistance in order to select treatment according to the aetiology. This may minimise the spread of infection, the progression to complications, and the development of antimicrobial resistance.

### Key messages

This study confirms that azithromycin 500 mg day 1 followed by 250 mg once daily in days 2–5 is an effective treatment of *M. genitalium* infections.

Azithromycin 1 g single dose leads to development of macrolide resistance in *M genitalium* and should be avoided as treatment of *M. genitalium*, chlamydia and urethritis/cervicitis of unknown aetiology.

Surveillance of *M. genitalium* macrolide resistance is recommended as rapid changes in the prevalence may occur.

Moxifloxacin is an effective second-line treatment of *M. genitalium* but development of resistance is a major concern.
